# Evaluation of Serum TNF-α and TGF-β in Patients with Oral Lichen Planus

**DOI:** 10.5681/joddd.2012.029

**Published:** 2012-11-12

**Authors:** Ali Taghavi Zenouz, Firoz Pouralibaba, Zohreh Babaloo, Masoumeh Mehdipour, Zahra Jamali

**Affiliations:** ^1^Associate Professor, Department of Oral Medicine, Faculty of Dentistry, Tabriz University of Medical Sciences, Tabriz, Iran; ^2^Assistant Professor, Department of Oral Medicine, Faculty of Dentistry, Tabriz University of Medical Sciences, Tabriz, Iran; ^3^Associate Professor, Department of Immunology, Faculty of Medicine, Tabriz University of Medical Sciences, Tabriz, Iran; ^4^Assoicate Professor, Department of Oral Medicine, Faculty of Dentistry, Shahid Beheshti University of Medical Sciences, Tehran, Iran

**Keywords:** Oral lichen planus, TGF-α, TNF-β

## Abstract

**Background and aims:**

The role of cytokines in the immunopathogenesis of oral lichen planus (OLP) has received much attention. The aim of this study was to evaluate the serum levels of TNF-α and TGF-β in patients with OLP in an Iranian population.

**Materials and methods:**

Thirty-two patients with OLP and 32 age-matched healthy volunteers as a control group were included in this study. Serum tests including TNF-α and TGF-β was performed in both groups. Data analysis was performed using descriptive statistics and Mann–Whitney U test using SPSS software version 16.0.

**Results:**

The mean of TNF-α in study and control groups were 157 ± 115 pg/ml and 14 ± 10 pg/ml, respectively. The difference between the two means was statistically significant (P < 0.001). Moreover, the mean of TGF-β in study and control groups were 155 ± 26 pg/ml and 175 ± 57 pg/ml, respectively. The difference between the two means was statistically significant (P = 0.03).

**Conclusion:**

According to the results of the present study, there was a significant decrease in the serum levels of TGF-β and a significant increase in the serum levels of TNF-α in patients with oral lichen planus. The increase in TNF-α serum levels in patients with OLP explains the inflammatory process in the course of the disease.

## Introduction

Lichen planus is a relatively common chronic mucocutaneous lesion with an unknown etiology, although several factors have been reported to involve in its etiology, including stress, hepatitis C, diabetes, trauma, genetic predisposition, and viral infections.^1,2^

A predominantly T lymphocyte infiltration in the subepithelial region is a histologic characteristic of lichen planus, which induces the release of chemokines and cytokines from T-helper 1 and T-helper 2 cells—these have a great role in the progression or recovery of the disease. In recent years, there has been increasing interest in the role of various cytokines in the immunopathogenesis of lichen planus, which include interleukin-2 (IL-2), interleukin-6 (IL-6), interleukin-10 (IL-10), tumor necrosis factor-α (TNF-α), and transforming growth factor-β (TGF-β).^3,4^

In a study on the salivary levels of interferon-γ (IFN-γ) and interleukin-4 (IL-4) in a group of patients with oral lichen planus, the results indicated a significant increase in the levels of both cytokines.^5^ Another study showed that TGF-β suppresses the immune response to auto-antigens, and the severity of inflammation from lichen planus increases in areas with reduced TGF-β activity.^6^

TGF-β has various functions, including growth inhibition, induction of apoptosis, effect on extracellular matrix structure and immunosuppression. It has also been demonstrated that TGF-β can increase the release of vascular endothelial growth factor (VEGF) in addition to having a significant anti-inflammatory effect.^7^ Another important cytokine is TNF-α, which has a role in the host immune response to infection, angiogenesis induction, tissue repair and regeneration, regulation of proliferation and tissue differentiation.^7,8^

According to a previous study,^8^ serum levels of TNF-α are significantly higher in patients with oral lichen planus compared to those in healthy controls. The serum levels of TGF-β were also significantly higher in patients with the erosive form compared to those with the reticular form. However, no statistically significant differences were found in TGF-β levels between patients with oral lichen planus and healthy controls. The results of another study indicated an increase in the salivary levels of TNF-α in patients with oral lichen planus; in addition, a significant relation was noted between TNF-α levels and different forms of oral lichen planus.^3^ TNF-α levels were higher in atrophic and erosive forms compared to the reticular form. Also, an immune response has been observed against TNF-α in 17 out of 22 patients with oral lichen planus.^9^ However, in the tissue samples of healthy controls a negative result was observed for TNF-α. Therefore, it was reported that TNF-α might have a role in the pathogenesis of oral lichen planus.^9^

Despite already published literature, there is still a limited number of studies that have evaluated the role of TNF-α and TNF-β in recent years and the experimental data are rather controversial with no general agreement. The aim of this study was to evaluate the serum levels of these two cytokines in patients with oral lichen planus and compare them with those in a group of healthy controls.

## Materials and Methods

The subjects consisted of patients, aged 18-60 years, with oral lichen planus, who were selected based on clinical diagnosis (diagnosis of the reticular form of lichen planus does not require biopsies or pathologic evaluations) or clinical-pathologic diagnosis from the patients referring to the Department of Oral Medicine, Faculty of Dentistry, Tabriz University of Medical Sciences.

Thirty-two subjects were selected by simple sampling method during a nine-month period. The controls consisted of 32 healthy individual, without lichen planus, who lived in the same geographic location and had presented to the same center.

After taking an accurate medical history and completing a preliminary checklist and taking biopsies (if necessary), TNF-α, and TGF-β serum level tests were administered. ELISA kit (Koma Biotech, Seoul, South Korea) was used to this end. Briefly, the specific human anti-TNF-α and anti-TGF-β antibodies were placed in the wells of each row of plates. The samples, including standard samples of the kit with known concentrations of TNF-α and TGF-β, the control samples and the sera of the subjects were introduced into these wells. During the initial incubation, TNF-α and TGF-β bind to the fixed antibody after irrigation, the specific biotin-bound antibodies for TNF-α and TGF-β are incorporated. During the second incubation phase, this antibody binds to TNF-α and TGF-β, which have been fixed during the initial incubation. After three irrigation procedures, streptovidin-bound peroxidase was added, which bound to the biotin-bound antigenic agent. After the third incubation procedure and irrigation to remove unbound enzymes, a chromogen substrate is incorporated, which reacts with the bound enzyme and produces color. The intensity of the light produced is directly proportional to TNF-α and TGF-β concentrations in the initial sample.

In the control subjects, CBC, FBC, HDL, LDL, TG and cholesterol tests, which are all considered screening tests, were ordered along with TNF-α and TGF-β serum level tests to encourage cooperation and to contribute to the evaluation of the health status of the subjects.

All subjects gave written informed consent to participate in the study. The study protocol and procedures were approved by the Research Ethics Committee of Tabriz University of Medical Sciences.

Patients with the following conditions were excluded from the study:

- Presence of any stimulus leading to lichenoid reactions, including use of any medications such as anticholinerigic agents, saliva-reducing agents, antihistamines, antihypertensives and adrenergic Beta-blockers during the past 6 months.
- Presence of any congenital or acquired immune system disorders, such as AIDS, or a history of chemotherapy, injection addictives, and individuals undergoing dialysis.
- Presence of signs and symptoms of systemic infections, allergies, smoking and use of medications for the above-mentioned conditions.

### Statistical Analysis

Data were analyzed with descriptive statistical methods (frequency, percentage and mean ± standard deviation), and Mann–Whitney U test using SPSS software version 16.0. Statistical significance was defined at P < 0.05.

## Results

Fisher’s exact test did not reveal any significant differences between the study and control groups in relation to gender (P = 0.45).

**Figure 1:**
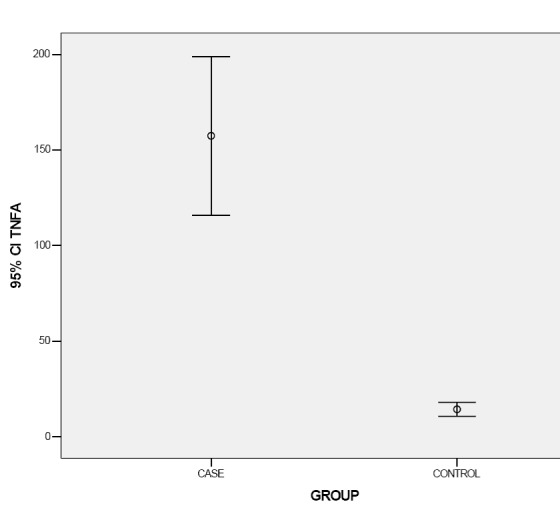
Means of TNF-α serum levels in the case (oral lichen planus) and control groups.

Means of TNF-α serum levels were 157.41 ± 115.30 pg and 14.44 ± 10.16 pg in the case and control groups, respectively. Mann–Whitney U test revealed statistically significant differences in TNF-α serum levels between the two groups (P < 0.001; Figure 1).

**Figure 2:**
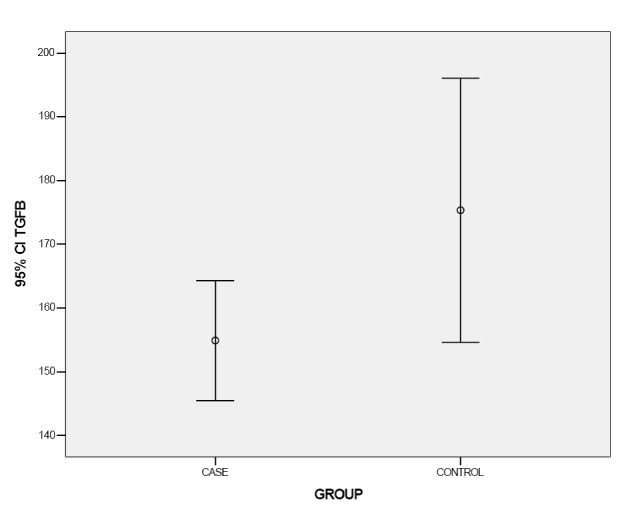
Means of TGF-β serum levels in the case (oral lichen planus) and control groups.

Means of TGF-β serum levels were 154.91 ± 26.08 pg and 175.34 ± 57.46 pg in the case and control groups, respectively. Mann–Whitney U test also revealed statistically significant differences in TNF-β serum levels between the two groups (P = 0.037; Figure 2).

## Discussion

In the present case-control study, the relationship between lichen planus and the serum levels of TNF-α and TGF-β was evaluated in two matched case and control groups.

The results showed a significant increase in the serum levels of TNF-α in OLP patients (P < 0.001). This finding emphasizes the role of this cytokine in the occurrence of autoimmune diseases including lichen planus. The results of a large number of studies have also confirmed the role of this key cytokine in inducing OLP.

TNF-α is involved in the secretion and initiation of the activity of matrix metalloproteinases of T-cells, which destroy the basal layer membrane proteins in conjunction with kimase and tripase secreted by mast cells.^10^ In addition, TNF-α induces apoptosis in a number of cells. Furthermore, some studies have shown that this cytokine induces the necrosis of epithelial cells, reducing the thickness of the epithelium.^10,11^ Therefore, TNF-α might be considered a factor involved in the incidence of ulcerations in and atrophy of the epithelium in OLP.

Rhodus et al^12^ found significantly high levels of nuclear factor-kappa B dependent cytokines (TNF-α, IL-1-α, IL-6, and IL-8) in tissue transudate of OLP patients. Pezelj-Ribaric et al^3^ also showed increased salivary levels of TNF-α in OLP. The observation of significantly higher TNF-α serum levels in OLP patients assessed in the present study is in line with these findings.

As mentioned earlier, TGF-β levels in the present study were significantly lower in OLP patients compared to healthy individuals (P < 0.037). T-cells secreting TGF-β have been identified and named Th-3 cells as a distinct population of antigen-specific CD4 T-cells. There is evidence that these cells inhibit the response of immune response to auto-antigens and prevent autoimmune reactions.^10,12,13^ TGF-β inhibits proliferation and differentiation of T-cells, activation of macrophages, and secretion of pro-inflammatory cytokines.^10^

TGF-β induces the synthesis of extracellular matrix proteins such as collagen and cellular receptors for matrix proteins such as integrins. These functions result in tissue regeneration after controlling local inflammatory and immunologic reactions.^7,14,15^

A decrease in TGF-β levels makes the individual susceptible to autoimmune lymphocytic inflammation while its administration in autoimmune disorders, in which T-cells have a prominent role, might have a therapeutic effect.^14,16^

The decrease in TGF-β levels in the present study might be attributed to depletion of TGF-β-secreting T-cells (Th-3) or regulatory T-cells, inhibition of TGF-β secretion, secretion of non-functional TGF-β, inadequate or incomplete expression of TGF-β receptors, and inadequate intracellular signals from TGF-β receptors. Zhou et al^8^ reported significantly higher serum levels of TNF-α in patients with OLP compared to healthy controls and higher serum levels of TGF-β in the erosive form of the disease compared to the reticular form. However, the overall serum levels of TGF-β in OLP patients were not significantly different from those in the controls,^8^ which is not consistent with the results of the present study. Considering this discrepancy, it should be noted that a decrease in TGF-β levels is consistent with the pathogenesis of the disorder. The different results of the latter study which do not coincide with the course of the disorder might be attributed to the fact that the etiology and pathogenesis of the erosive form of lichen planus may be different from other forms of the disorder. It is also possible that the TGF-β secreted is non-functional or it does not have sufficient number of specific receptors; however, further studies are necessary in this regard.^7,16^

Finally, it should be pointed out that OLP is a multifactorial disorder with an unknown etiology and several risk factors have been suggested as its etiologic agents and TGF-β is only one of them. On the other hand, TGF-β has been known to have several roles in the etiology of OLP and it is possible that a change in its serum level cannot be considered as the sole etiologic factor. It is suggested that the counts and function of regulatory T-cells be evaluated in oral lichen planus patients.

TNF-α and TGF-β serum levels be compared in patients with the atrophic, erosive and reticular forms of the disorder.

The relationship between serum levels of TNF-α and TGF-β and the clinical severity of the disorder and the response to treatment be evaluated.

## Conclusion

Significant changes were evident in the serum levels of TNF-α and TGF-β in patients with oral lichen planus. The increase in TNF-α serum levels in patients with OLP may, in part, explain the inflammatory process in the course of the disease. The results of the present study indicate a decrease in the factors controlling the autoimmune reactions, i.e. the activity of regulatory T-cells, which are the principle cells to secrete TGF-β. Therefore, it can be concluded that a decrease in the activity of regulatory T-cells might have a role in the pathogenesis of OLP.
